# Developing a Machine‐Learning Predictive Model for Retention of Posterior Cruciate Ligament in Patients Undergoing Total Knee Arthroplasty

**DOI:** 10.1111/os.14076

**Published:** 2024-05-01

**Authors:** Long Chen, Liyi Zhang, Diange Zhou, Shengjie Dong, Dan Xing

**Affiliations:** ^1^ Arthritis Clinical and Research Center Peking University People's Hospital, Peking University Beijing China; ^2^ Orthopedic Department Beijing Jishuitan Hospital Beijing China; ^3^ Orthopedic Department Yantaishan Hospital Yantai China

**Keywords:** Knee joint, Machine learning, Posterior cruciate ligament, Total knee arthroplasty

## Abstract

**Objective:**

Predicting whether the posterior cruciate ligament (PCL) should be preserved during total knee arthroplasty (TKA) procedures is a complex task in the preoperative phase. The choice to either retain or excise the PCL has a substantial effect on the surgical outcomes and biomechanical integrity of the knee joint after the operation. To enhance surgeons' ability to predict the removal and retention of the PCL in patients before TKA, we developed machine learning models. We also identified significant feature factors that contribute to accurate predictions during this process.

**Methods:**

Patients' data on TKA continuously performed by a single surgeon who had intended initially to undergo implantation of cruciate‐retaining (CR) prostheses was collected. During the sacrifice of PCL, we utilized anterior‐stabilized (AS) tibial bearings. The dataset was split into CR and AS categories to form distinct groups. Relevant information regarding age, gender, body mass index (BMI), the affected side, and preoperative diagnosis was extracted by reviewing the medical records of the patients. To ensure the authenticity of the research, an initial step involved capturing X‐ray images before the surgery. These images were then analyzed to determine the height of the medial condyle (MMH) and lateral condyle (LMH), as well as the ratios between MLW and MMH and MLW and LMH. Additionally, the insall‐salvati index (ISI) was calculated, and the severity of any varus or valgus deformities was assessed. Eight machine‐learning methods were developed to predict the retention of PCL in TKA. Risk factor analysis was performed using the SHApley Additive exPlanations method.

**Results:**

A total of 307 knee joints from 266 patients were included, among which there were 254 females and 53 males. A stratified random sampling technique was used to split patients in a 70:30 ratio into a training dataset and a testing dataset. Eight machine‐learning models were trained using data feeding. Except for the AUC of the LGBM Classifier, which is 0.70, the AUCs of other machine learning models are all lower than 0.70. In importance‐based analysis, ISI, MMH, LMH, deformity, and age were confirmed as important predictive factors for PCL retention in operations.

**Conclusion:**

The LGBM Classifier model achieved the best performance in predicting PCL retention in TKA. Among the potential risk factors, ISI, MMH, LMH, and deformity played essential roles in the prediction of PCL retention.

## Graphical abstract

Data from 307 knee joints of 266 total knee arthroplasty (TKA) patients, including 5 categorical and 7 continuous variables, was used for training 8 machine types to predict PCL removal and retention. The best model, LGBM classifier, was selected by AUC method, highlighting insall‐salvati index (ISI), lateral condyle height (LMH), and medial condyle height (MMH) as key variables.

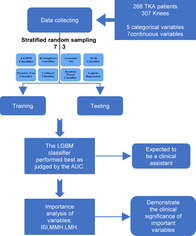

## Introduction

According to a study of primary total knee arthroplasty (TKA) procedures, 38% used a cruciate retaining (CR) construct, while 53% used a posterior cruciate ligament (PCL) substituting (PS) prosthese or condylar stabilized construct.^1^ However, there has been an ongoing debate regarding the optimal approach for achieving a stable knee that will improve the patient's quality of life, specifically whether a PCL‐retaining or substituting approach is better.^2^ Both forms of prosthesis have their advantages and disadvantages. Previous studies have shown that there are no significant differences in postoperative functional outcomes between the two technologies.^2^, ^3^ However, it was observed that abnormal anterior translation occurred in low and mid flexion after removing PCL, and the normal motion was not restored by the PS prosthesis.^4^ While prosthesis choice has historically been based on surgeon preference and individual patient characteristics,^5^ it is crucial to base surgical choices on evidence‐based summaries of available information rather than relying solely on the surgeon's personal experience or preference.^5^ This approach ensures that decisions are made objectively and in line with the best practices established by scientific research.

Under physiological conditions, the PCL plays a crucial role in maintaining the stability of knee flexion, varus and valgus. Retaining the PCL in knees can lead to improved proprioception and kinematics, better bone preservation, reduced load between bones, and improved implant stabilization.^6^ On the other hand, potential advantages of PCL substituting knees include a less technically demanding procedure, better knee flexion, more predictable kinematics, and a more stable component interface.^5^, ^7^ The two prosthesis operations differ not only in concept but also in technique. Performing CR TKA can be challenging in specific situations, such as when there is PCL insufficiency, severe deformity, a history of trauma, or prior surgical procedures.^8^ Individuals with pronounced flexion contracture, an inclined posterior slope, and a comparatively diminutive femoral component encounter an increased susceptibility to necessitating intraoperative conversion from CR TKA to PS TKA.^5^ If the integrity of the PCL cannot be guaranteed during surgery, it becomes necessary to switch to PS TK. Thus, some potential anatomical and demographic factors may affect PCL retention during TKA.^5^ Surgeons should possess knowledge of potential risk factors associated with PCL sacrifice and should have sufficient evidence to justify their decision‐making.

Taking all those factors into consideration, choosing the appropriate prosthesis type is crucial for the success and effectiveness of the operation, and it is necessary to have a predictive method in place.^9^ However, the current research on prediction methods is insufficient. Unfortunately, many surgeons still rely on dogmatic preconceptions or rigid preferences when selecting a prosthesis. Therefore, we utilized diverse machine learning methods, taking the socio‐demographic and imaging data of patients prior to surgery as variables to predict the retention of the final PCL. We conducted a retrospective analysis using data from 24 consecutive months of TKA surgery performed by a single surgeon at our hospital, predicting the outcome in terms of the type of surgery that was ultimately executed.

This research aimed to achieve three objectives: (i) Establish machine learning predictive models using preoperative patient data to predict the preservation of the PCL. (ii) Identify the most suitable predictive model for accurately forecasting the necessity of preserving the PCL. (iii) Identify key predictive factors that play a significant role in the prediction process, providing valuable insights for clinical predictions and aiding surgeons in making informed decisions about PCL preservation. Understanding these factors will enhance predictive accuracy and illuminate the biomechanical and clinical considerations influencing PCL preservation decisions in TKA.

## Materials and Methods

### Inclusion and Exclusion Criteria

The study inclusion criteria were: (i) Patients who underwent primary TKA at the present hospital from January 2019 to August 2021. (ii) The primary TKAs were operated by the same surgeon using the same prosthesis (Zimmer Biomet, Vanguard CR) and were allowed intraoperative sacrifice of the PCL (Zimmer Biomet, Vanguard AS). All TKAs were prepared for CR prostheses by the same surgeon. At the same time, data with missing values in the variable of interest were excluded.

### Machine Learning Development Process Data Preprocessing

**Figure 1:**
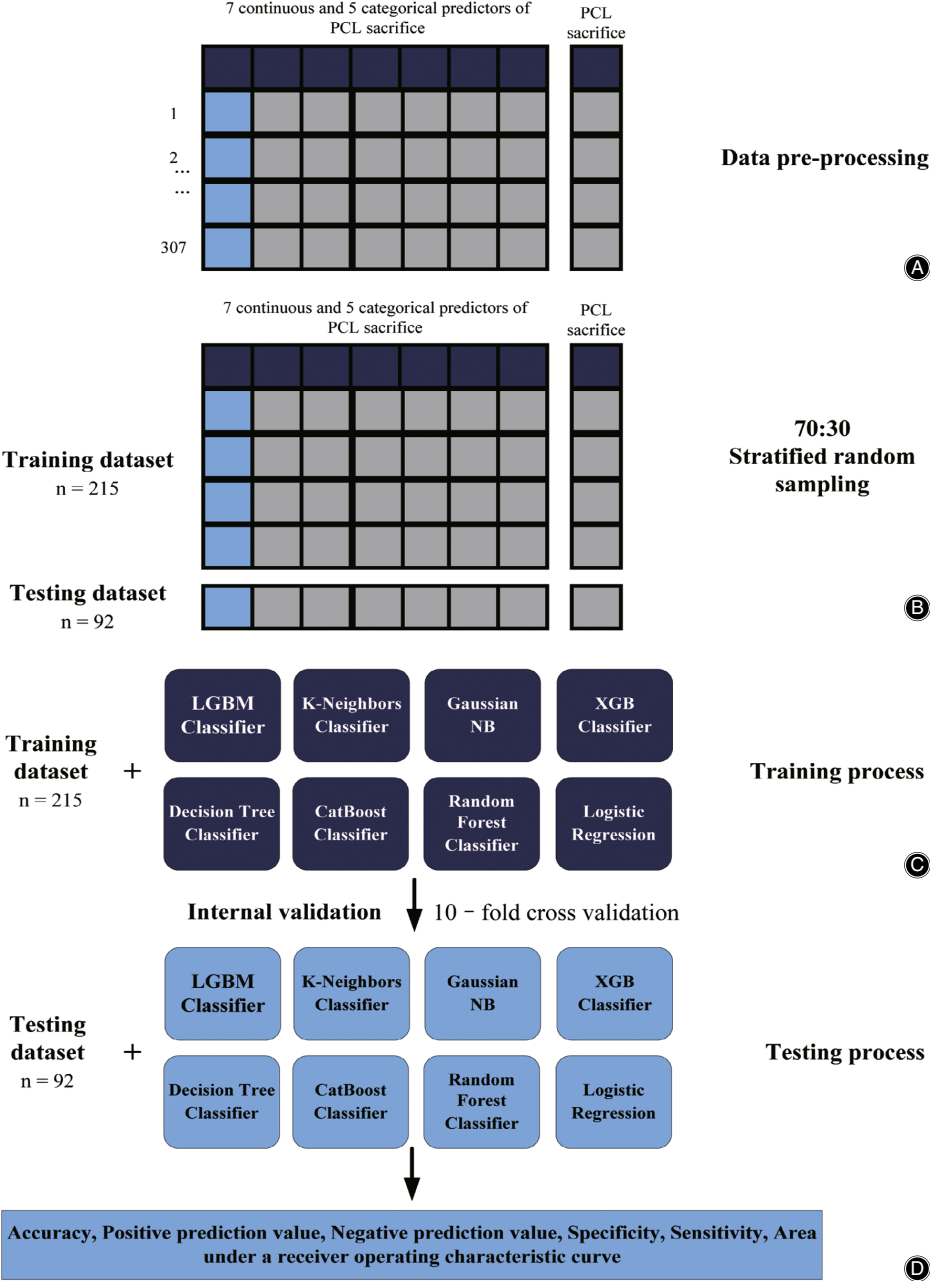
Machine learning development process (A) 7 continuous and 5 categorical predictors of PCL retention were taken into the computational process. (B) A stratified random sampling technique was applied to split patients in a 70:30 ratio to a training dataset and a testing dataset. (C) Training dataset was used to identify the optimal hyperparameters which provided the highest accuracy in a tenfold internal cross‐validation of each model. (D) The performance of all algorithms was evaluated with another, unseen, testing dataset.

The data set for our study consisted of a total of 262 patients with 12 variables. These variables comprised 7 continuous variables and 5 categorical variables. The continuous variables were age, body mass index (BMI), medial condyle height (MMH), lateral condyle height (LMH), medial‐lateral width of epicondyle (MLW), the ratio of MLW and MMH, and the ratio of MLW and LMH. The categorical variables were sex, preoperative diagnosis, side, Insall‐salvati index (ISI), and lower‐extremity mechanical axis. The calculation process for PCL sacrifice incorporated 7 continuous predictors and 5 categorical predictors using standard dummy coding (Figure 1). There were no missing data in the dataset.

**Figure 2:**
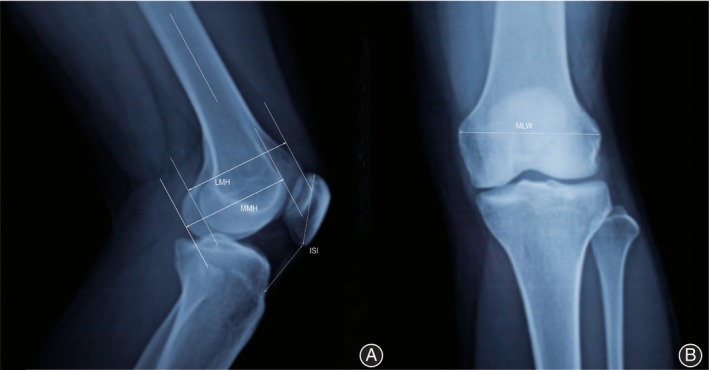
(A) A lateral X‐ray of the knee joint illustrated the measurement process of the medial condyle height (MMH), lateral condyle height (LMH), and Insall‐salvati index (ISI). (B) An anteroposterior X‐ray of the knee joint demonstrated the measurement process of the width of the epicondyle (MLW).

Age, gender, BMI, preoperative diagnosis, and side were obtained by reviewing the patients' medical records. Radiographic data was obtained through preoperative X‐ray films (Figure 2). The MLW was measured using the anterior view of the knees.^10^ The measurement of MMH and LMH was conducted by employing the lateral view of the knees, while a full limb radiograph was utilized to measure the mechanical axis.

In order to classify the lower‐extremity mechanical axis, cut‐offs of 15° varus, 5° varus, 5° valgus, and 15° valgus were utilized to distinguish between severe varus deformity, mild varus deformity, neutral position, mild valgus deformity, and severe valgus deformity. Additionally, the ISI was divided into four quartiles.

### Algorithm Training and Validation

The patients were divided into a training dataset and a testing dataset using the stratified random sampling technique, maintaining a 70:30 ratio.^11^ Eight machine learning models were constructed to predict PCL preservation during TKA, including KNeighbors Classifier, Gaussian MB, XGB Classifier, LGBM Classifier, Decision Tree Classifier, CatBoost Classifier, Random Forest Classifier, SVC, and Logistic Regression.

To better understand how each feature impacts model predictions, we introduced the SHApley Additive exPlanations (SHAP).^12^ This approach constructs an additive explanatory model where all features are treated as contributors. By using the SHAP toolkit, it is possible to gain insights into how each feature influences the prediction results.

To assess the performance of machine learning models, the measurement of the area under the receiver operating characteristic curve (AUC) was carried out.^13^ The AUC is a crucial metric for assessing the performance of machine learning predictive models, particularly in binary classification. AUC reflects the probability that the model will rank a randomly chosen positive instance higher than a randomly selected negative one, providing a comprehensive evaluation of the model's ability to distinguish between the two classes. Throughout these procedures, the programming language Python (version 3.9.0; from Python Software Foundation, located in Wilmington, DE, USA) and Scikit‐Learn (version 1.0.2, a library for Machine Learning) were employed.

### Statistical Analysis

A comparative analysis was undertaken to evaluate various continuous variables, namely Age, BMI, MLW, LMH, the ratio of MLW to MMH, and the ratio of MLW to LMH, between the CR and AS groups. This comparison was conducted using Student's *t*‐test. Moreover, categorical variables such as sex, preoperative diagnosis, lower‐extremity mechanical axis, and the ISI were analyzed between the CR and AS groups using the chi‐squared test. All statistical analyses were performed using SPSS software (Version 26.0, IBM® Chicago, IL, USA). A significance level of less than 0.05 on a two‐tailed test was considered statistically noteworthy.

## Results

### Baseline Characteristics

We ultimately incorporated a total of 307 knees obtained from 266 patients. Among these individuals, 254 were female, while the remaining 53 were male participants. The age range of the patients spanned from 33 to 90 years, with the average age at the time of TKA being 67.02 ± 7.57 years. Prior to the surgical procedure, 297 cases of osteoarthritis and 10 cases of rheumatoid arthritis were identified through diagnosis. To compile patients' data, information regarding age, gender, body mass index (BMI), preoperative diagnosis, as well as other demographic and clinical characteristics, were extracted from their medical records (Table 1).

**Table 1:** Comparison of the demographic and clinical characteristics of all patients

Patient characteristics	AS group	CR group	*T*/*χ* ^2^	*p* value
Gender, *n* (%)			0.045	0.833
Female	73 (23.7)	181 (58.9)		
Male	16 (5.2)	37 (12.0)		
Age (years), mean ± SD	66.17 ± 8.03	67.37 ± 7.37	−1.259	0.209
BMI (kg/m^2^), mean ± SD	26.49 ± 3.76	26.87 ± 3.56	−0.840	0.402
Preoperative diagnosis, *n* (%)			8.445	0.008
Rheumatoid	7 (2.2)	3 (0.9)		
Osteoarthritis	82 (26.7)	215 (70.0)		
Side, *n* (%)			2.688	0.101
Left	50 (16.2)	100 (32.5)		
Right	39 (12.7)	118 (38.4)		
MMH (CM), mean ± SD	6.28 ± 0.63	6.33 ± 0.58	−0.586	0.558
LMH (CM), mean ± SD	6.25 ± 0.58	6.30 ± 0.53	−0.666	0.506
MLW (CM), mean ± SD	8.40 ± 0.74	8.37 ± 0.69	0.365	0.715
The ratio of MLW and MMH	0.75 ± 0.06	0.76 ± 0.06	1.113	0.258
The ratio of MLW and LMH	0.75 ± 0.05	0.75 ± 0.05	1.433	0.153
Insall‐salvati index, *n* (%)				
0–1/4	36 (11.7)	41 (13.3)		
1/4–2/4	20 (6.5)	56 (18.2)		
2/4–3/4	15 (4.8)	60 (19.5)		
3/4–1	18 (5.8)	61 (19.8)		
*p* for trend			16.488	0.001
Lower‐extremity mechanical axis, *n* (%)				
>15° varus	20 (6.5)	25 (8.1)		
Varus between 5° to 15°	43 (14.0)	137 (44.6)		
Neutral position	15 (4.8)	42 (13.6)		
Valgus between 5° to 15°	7 (2.2)	10 (3.2)		
Valgus >15°	4 (1.3)	4 (1.3)		
*p* for trend			10.636	0.027

We employed femoral components spanning from 55 to 72.5 mm in size, along with tibial components ranging from 63 to 83 mm in size. The femoral components varied in their medial‐lateral lengths, corresponding to the different sizes employed, ranging from 59 to 75 mm. Furthermore, polyethylene inserts with thicknesses of 10, 12, and 14 mm were utilized. The groups exhibited no discernible differences concerning the sizes of the tibial and femoral components or the substitution of the patella (Table 2).

**Table 2:** Comparison of implanted components between AS and CR.

Component characteristics	AS group	CR group	χ^2^	*P* value
The size of the tibial component			8.520	0.074
63 mm	13	13		
67 mm	76	26		
71 mm	86	32		
75 mm	28	9		
79/83 mm	15	10		
The size of the femoral component			9.624	0.150
55 mm	8	9		
57.5 mm	66	20		
60 mm	73	26		
62.5 mm	31	16		
65 mm	14	10		
67.5 mm	19	7		
70/72.5 mm	7	2		
The thickness of the polyethylene insert			9.012	0.003
10 mm	171	56		
12 and 14 mm	47	34		
Patella replacement (Yes/No)	122/95	47/43	0.254	0.614

### Machine‐Learning Performance Comparisons

**Figure 3:**
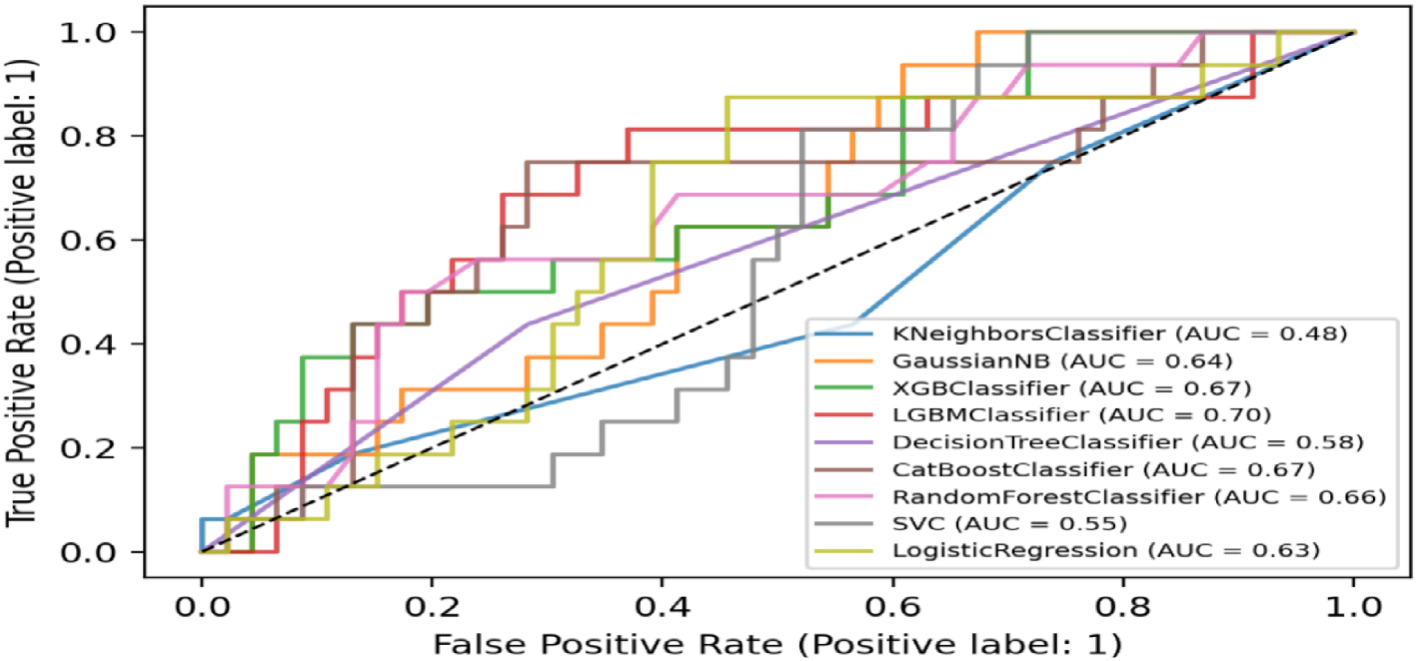
Receiver‐operating characteristic curve (ROC) for the models developed with all algorithms. Except for the LGBM Classifier's 0.70, the AUCs of other machine learning models were all lower than 0.70.

We developed eight machine‐learning models to predict whether the PCL could be retained successfully. Figure 3 shows the receiver operating characteristic curve for the models, which were created using all variables as inputs. The AUC value for each model is labeled on the graph. Except for the LGBM Classifier model, which had an AUC of 0.70, all other models had an AUC lower than 0.70. There was 0.48 for the KNeighbors Classifier, 0.64 for Gaussian MB, 0.67 for the XGB Classifier, 0.58 for the Decision Tree Classifier, 0.67 for the CatBoost Classifier, 0.66 for the Random Forest Classifier, 0.55 for the SVC and 0.63 for the Logistic Regression (Figure 3).

### Machine‐Learning‐Model Selection

The LGBM Classifier model surpassed alternative models regarding accuracy, positive predictive value, and sensitivity (Figure 3). Additionally, the model had a higher AUC and good calibration. As a result, the LGBM Classifier algorithm was selected for model construction and was deemed the most effective in predicting whether PCLs could be retained in TKA.

Leveraging LGBM's advanced tree‐based algorithm, which excels in processing large‐scale data and capturing complex patterns through its efficient ‘Leaf‐wise’ split approach and direct handling of categorical features, our aim was to enhance surgical decision‐making. This model is distinguished by its high computational efficiency, accuracy, and capability to address overfitting through extensive regularization options.^14^ As a result, it is particularly well‐suited for predicting surgical outcomes in TKA procedures, which require nuanced analysis and involve a significant amount of data.

### Analysis of Clinical Variable Contribution

**Figure 4:**
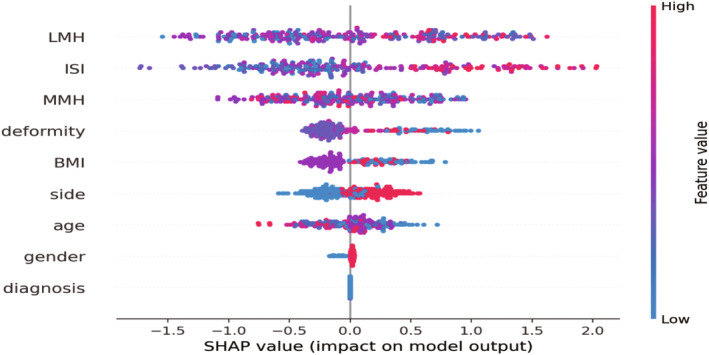
Characteristics of the selected model (LGBM Classifier): SHAP Value summary graph of top variables and their impact on the prediction.

**Figure 5:**
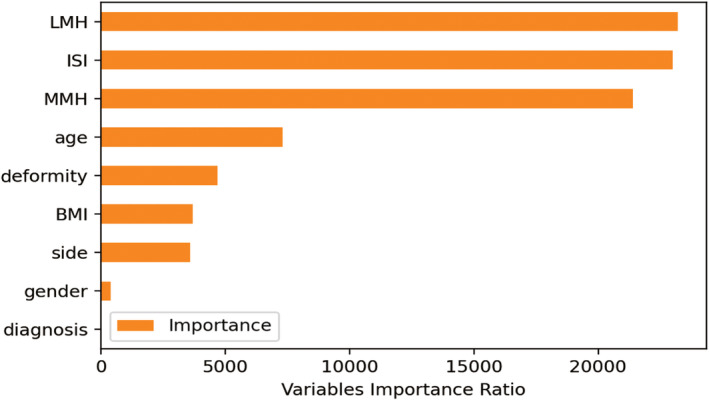
Variables importance ratio. Top nine crucial variables on the prediction of posterior cruciate ligament preservation.

In the field of explainability based on feature importance, it was crucial to understand the significant features that our classifier considered when making its final decisions. Figure 4 presented a sorted list of features for the classifier responsible for predicting sacrifices, which demonstrated superior performance. Then, we utilized the SHAP explainer to calculate the importance of features. The subsequent features held substantial influence over the final predictions of the model: LMH, ISI, MMH, deformity, BMI, side, age, gender, and preoperative diagnosis. The scatter plot depicting feature rankings, based on the sum of the average absolute values of SHAP, provided a visual representation of all the samples. The critical predictive factors for PCL retaining were confirmed to be LMH, ISI, MMH, and deformity, as shown in Figure 4. Additionally, Figure 5 showcased the Variables' Importance Ratio graph, which highlighted the order of importance for variables and their impact on the predictions made by the LGBM Classifier model. The results indicated a strong consistency between the key predictive variables identified through the Variable Importance Ratio and those explained by the SHAP explainer. Therefore, this alignment highlighted the trustworthiness and accuracy of the model's understanding of the factors that greatly impacted its predictions.

## Discussion

### Comparison of Models

This retrospective study utilized machine learning techniques, such as Random Forest Classifier, CatBoost Classifier, LGBM Classifier, and XGB Classifier, to evaluate the risk of sacrificing PCL. Among these methods, the LGBM Classifier approach demonstrated superior performance, with the highest AUC of 0.70 reported by this model. According to feature importance‐based analysis, we found that patients with lower ISI, smaller LMH, and MMH had a higher risk of sacrificing PCL in a preoperatively planned CR TKA.

### Condition of the PCL

It is important to note that the condition of the PCL can have a significant impact on various postoperative factors, including range of motion, stability, forces at the interface between the bone and prosthesis, femoral rollback, gait pattern, wear, and proprioception.^15^ If the PCL is too loose, it may result in instability and knee pain. On the other hand, excessive tension in the PCL can restrict flexion and increase stress, leading to potential wear of the polyethylene component.^16^ Therefore, achieving appropriate PCL balance is crucial in order to optimize knee flexion and achieve favorable clinical outcomes following CR TKA.^17^ In this particular study, the tension of the PCL during CR TKA was determined by the same surgeon. The decision to sacrifice PCL was made in TKA based on the PCL tightness, PCL damage, and other associated clinical situations.

### Several Important Predictors

Some studies have shown that ISI, most commonly used to determine patellar height, is an essential predictor of sacrificing PCL.^18^ Patients with a higher position of the patella tend to retain the PCL compared to individuals with a lower patellar position.^10^ According to the investigation conducted by Wang *et al*., it was discovered that an increased patellar position corresponded to a decreased patellar tendon strain. This decrease in strain facilitates achieving an appropriate joint separation.^10^ As the knee flexes to a 90° angle, the PCL and patellar tendon align closely with the longitudinal axis of the tibia. Hence, the patellar tendon plays a crucial role in preserving the joint gap during knee flexion. Our recent research solidified the findings that patients with an elevated patellar position were more likely to retain intact PCL.

MMH and LMH were referred to as anteroposterior diameter of the medial and lateral femoral condyle. Previous research has indicated that patients with ACL rupture exhibit a larger anteroposterior diameter of the lateral femoral condyle.^19^ It suggested that the anterior and posterior diameters of the femoral condyle have a significant impact on the stress distribution and contact area of the artificial knee joint. The larger the anterior and posterior diameter of the femoral condyle, the more stable the artificial knee joint, the larger the contact area, and the smaller the stress.^20^ It is not difficult to find that the anteroposterior diameter of the femoral condyle represented the size of the femoral condyle, which played an important role in the stability of the knee joint during flexion.^21^ We believed that the increased risk of sacrificing PCL might be due to the relatively smaller component sizes of the femoral condyle. Hence, the anteroposterior dimension of the medial and lateral condyles of the femur may serve as a valuable tool to achieve equilibrium in the flexion compartment during a CR TKA. Consequently, there would be no need to transition to a PS TKA.

In individuals suffering from a severe coronal deformity, it is expected to observe bone erosion or condylar dysplasia on the concave side of the deformity.^22^ On the other hand, tension forces acting on the convex side of the joint impact it extensively, leading to elongated soft tissues and subsequently complicating gap balancing. Consequently, the necessity of PCL resection arises. These findings support the notion that PCL sacrifice offers greater efficiency in achieving proper mediolateral balancing in TKA cases involving severe deformities. Thus, in the current study, the surgeon opted for AS insert usage over CR insert in the course of TKA.

### Application and Prospect

The study has successfully developed a machine learning predictive model to determine whether the PCL is retained during TKA. This model will enable surgeons to make more informed preoperative decisions, potentially reducing costs associated with trial and error during surgery. The predictive model is anticipated to improve clinical diagnostic and treatment processes significantly. This study highlights the importance of integrating advanced computational tools to enhance surgical outcomes and optimize patient care strategies. Continuous validation and iterative improvements are crucial for ensuring the model's effectiveness as it is used in the clinic, with the number of samples increasing and supported by feedback from surgeons.

### Limitations

This research presents several limitations worth considering. To begin with, it is important to acknowledge that this study adopts a retrospective design, which inherently brings forth its vulnerabilities and potential bias. Nonetheless, it is crucial to emphasize that the instances were identified utilizing highly comprehensive patient data acquired from a singular medical facility and with TKA carried out by a sole surgeon. The sample size of individuals who underwent PCL sacrifice in this study was limited, which may have reduced the statistical power and robustness of the findings. Increasing the number of categories for categorical variables could enhance the clinical applicability and relevance of our research. However, given the small sample size, expanding the range of categorical variables might compromise the effectiveness of the statistical tests employed. Second, the decision to convert to the AS insert was based on the technique and experience of a single surgeon, which may have introduced selection bias. The radiographic data in this study primarily came from X‐ray imaging, which is commonly used in clinical diagnostics and treatment in TKA. However, incorporating low‐dose computed tomography (CT) might be able to provide different perspectives and potentially influence the predictive results of our model in the future. Finally, we did not incorporate the clinical characteristics of patients into our model, which could contribute to another potential limitation. In order to build a more complete prediction model, more clinical and radiological factors need to be collected as predictors for prediction in the future.

## Conclusion

The present study highlighted that CR TKA may not always be feasible and provided insight into predicting PCL preservation using machine learning models. The LGBM Classifier model demonstrated the best performance for predicting retention of PCL in TKA patients. Additionally, we emphasized the importance of considering factors such as ISI, MMH, LMH, and deformity when determining the feasibility of CR TKA.

## Author Contributions

All authors had full access to the data in the study and took responsibility for the integrity of the data and the accuracy of the data analysis. Conceptualization, S.D. and D.X.; Methodology, S.D., D.X.; Investigation, L.Z., D.Z., and L.C.; Formal Analysis, L.C. and L.Z.; Resources, S.D. and D.X.; Writing ‐ Original Draft, L.C. and D.X.; Writing ‐ Review & Editing, L.C., S.D., D.X.; Visualization, S.D.; Supervision, D.X.; Funding Acquisition, D.X.

## Ethics Statement

The ethical application for the relevant studies was approved and authorized by the ethics committee of Peking University People's Hospital (2021PHB431‐001).

## Authorship Declaration

All authors listed meet the authorship criteria according to the latest guidelines of the International Committee of Medical Journal Editors. All authors are in agreement with the manuscript.
